# Heteroaromatic organic compound with conjugated multi-carbonyl as cathode material for rechargeable lithium batteries

**DOI:** 10.1038/srep23515

**Published:** 2016-04-11

**Authors:** Meixiang Lv, Fen Zhang, Yiwen Wu, Mujuan Chen, Chunfeng Yao, Junmin Nan, Dong Shu, Ronghua Zeng, Heping Zeng, Shu-Lei Chou

**Affiliations:** 1 School of Chemistry and Environment, Guangzhou Key Laboratory of Analytical Chemistry for Biomedicine, South China Normal University, Guangzhou 510006, China; 2 Research Resources Center, South China Normal University, Guangzhou 510006, China; 3 Institute for Superconducting and Electronic Materials, Australian Institute for Innovative Materials, University of Wollongong, Innovation Campus, Squires Way, North Wollongong NSW 2522, Australia

## Abstract

The heteroaromatic organic compound, *N,N’*-diphenyl-1,4,5,8-naphthalenetetra- carboxylic diimide (DP-NTCDI-250) as the cathode material of lithium batteries is prepared through a simple one-pot *N*-acylation reaction of 1,4,5,8-naphthalenetetra-carboxylic dianhydride (NTCDA) with phenylamine (PA) in DMF solution followed by heat treatment in 250 °C. The as prepared sample is characterized by the combination of elemental analysis, NMR, FT-IR, TGA, XRD, SEM and TEM. The electrochemical measurements show that DP-NTCDI-250 can deliver an initial discharge capacity of 170 mAh g^−1^ at the current density of 25 mA g^−1^. The capacity of 119 mAh g^−1^ can be retained after 100 cycles. Even at the high current density of 500 mA g^−1^, its capacity still reaches 105 mAh g^−1^, indicating its high rate capability. Therefore, the as-prepared DP-NTCDI-250 could be a promising candidate as low cost cathode materials for lithium batteries.

Rechargeable lithium batteries dominate the portable electronics market because lithium cells offer the largest energy density in all the rechargeable battery technologies available. Additionally, they are well-positioned to take over the emerging large-scale application markets of electric vehicles and off-grid storage devices^1^,^2^,^3^,^4^,^5^. The performance of rechargeable lithium batteries is mainly restricted by the cathode based on inorganic intercalation compounds with low specific capacity^6^,^7^. Recently, organic carbonyl compounds as high-energy cathode materials for rechargeable lithium batteries have been extensively explored owing to redox stability, structural diversity, high theoretical capacities, and infinite availability from biomass^8^,^9^,^10^,^11^,^12^,^13^,^14^,^15^,^16^,^17^,^18^. Nevertheless, organic carbonyl compounds suffer from some mortal shortages, such as the intrinsic drawback of poor cyclic performance and rate capability owing to low electronic conductivity, high solubility in organic electrolyte, no well-defined Li^+^-conducting channel, and no interstitial sites due to the lack of lattice in organic electrode materials. To address these issues, first, the combination of multi-carbonyl groups and low molecular weight should be used to achieve high specific capacity, where multi-carbonyl groups can capture more lithium to offer multi-electron reactions^8^,^19^. Second, the carbonyl-based electrode generally demands certain functional structures to stabilize the negatively charged carbonyl groups under the electrochemical reduction state. Carbonyl groups are directly connected to an aromatic core to disperse charge through delocalization^8^,^10^,^20^,^21^,^22^. Third, from the design of electronic conductivity for organic semiconductors, one method is the judicious incorporation of heteroaromatic structures. The morphological and electronic properties of the target compounds can be predictably tuned in a wide range by this method^19^,^23^,^24^,^25^. For example, pyridine is an important electron-withdrawing moiety in electron-transporting materials^26^,^27^. According to the above function-oriented design strategies of organic compounds, many carbonyl-based positive electrode materials with high capacity and good electrochemical performance have been reported. For example, Sun *et al.* reported 3,4,9,10-perylene-tetracarboxylicacid-dianhydride (PTCDA) sulfide polymers, which showed average reversible capacity of ~130 mAh g^−1^ in current density of 100 mA g^−1 ^10. Yao *et al.* prepared 2,5-dimethoxy-1,4-benzoquinone (DMBQ) as cathode material, the electrode material demonstrated an initial discharge capacity of 312 mAh g^−1^ at current density of 10 mA g^−1^, and the capacity decayed sharply with the increase of cycles^28^. Chen *et al.* synthesized two new carbonyl electrodes, pyrene-4,5,9,10-tetraone (PTO) and 1,10-phenanthroline-5,6-dione (PhenQ), delivering a reversible capacity of 360 mAh g^−1^ in current density of 0.05 C^8^. Tarascon *et al.* reported an organic salt, lithium 2,6-bis(ethoxycarb onyl)-3,7-dioxo-3,7-dihydro-*s*-indacene-1,5-bis (olate), delivering an overall capacity of 125 mAh g^−1 ^1. Poizot *et al.* obtained tetralithium salt of tetrahydroxybenzoquinone (Li_4_C_6_O_6_), the Li_4_C_6_O_6_ compound showed good electrochemical performance with a sustained reversibility of ~200 mAh g^−1 ^11. Our group previously reported a series of polycarbonyl organic compounds with different number of hydroxyl groups and a lithium-organic coordination compound, [Li_2_(C_14_H_6_O_4_)], to achieved high initial discharge capacities^29^,^30^. However, one of the great challenge for these organic compounds is that most of the synthesis procedure were involved by many pot reactions or in high-temperature, with long time consuming. Furthermore, there is extremely large space to design the low molecular weight compounds with more carbonyl groups, conjugated system and fused heteroaromatic structures, which can improve the capacity, cyclic stability and rate performance of small molecules.

In this work, we report a compound with multi-carbonyl groups, conjugated system and fused heteroaromatic structures, *N,N′*-diphenyl-1,4,5,8-naphthalenete tracarboxylic diimide (DP-NTCDI-250) through a simple one-pot *N-*acylation reaction of 1,4,5,8-naphthalenetetracarboxylic dianhydride (NTCDA) with phenylamine (PA) in DMF and Et_3_N solution. The key features of this method are time-saving and low energy consumption. It should be noted that DP-NTCDI has been reported in literature^31^,^32^,^33^,^34^,^35^, however, DP-NTCDI was not applied as electrode material for lithium batteries. Here, DP-NTCDI-250 (the heat treated sample of DP-NTCDI) was used as cathode material showing high discharge capacity, good cycling stability and rate capability, which is superior over other benzene-fused analogues^29^,^30^,^36^,^37^,^38^, showing a promising organic cathode materials for rechargeable lithium batteries.

## Results and Discussion

### Synthesis mechanism

**Figure 1:**
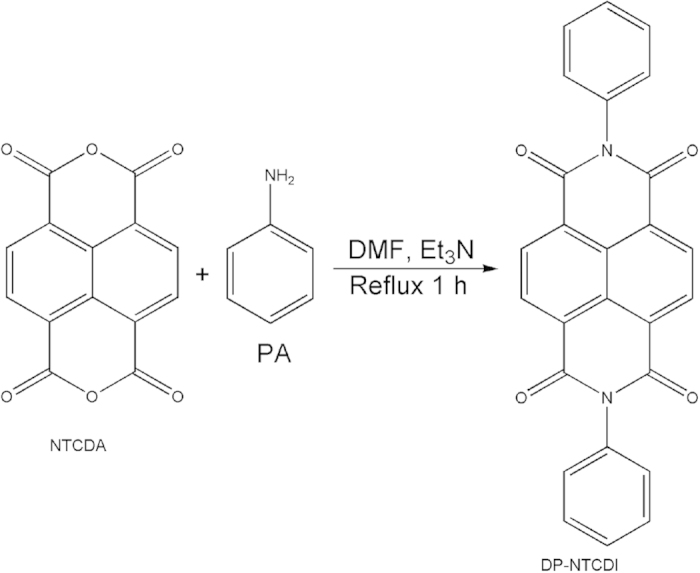
Synthesis of DP-NTCDI.

The synthesis of DP-NTCDI was adapted by one-pot *N*-acylation reaction (Fig. 1). NTCDA dissolved in DMF was reacted with PA, in which the -*N*- replaced the oxygen of -C-O-C- in NTCDA to form heteroaromatic compound, resulting in the generation of DP-NTCDI. The reaction was carried out in nitrogen atmosphere to avoid oxidation or decomposition of the PA. A brown precipitate, which can easily be separated by centrifugation and filtration, occurred as the reaction proceeds. It should be noted that the Et_3_N can act as catalyst and can be used to stimulate the reaction of NTCDA and PA. After washing with EtOH and recrystallizating in DMF and drying, the as-synthesized orange acicular product (DP-NTCDI), with a yield of 91.2%, was further heated at 250 °C to get DP-NTCDI-250 compound. The sample was prepared within 1 h through a simple one-pot *N*-acylation reaction. The key features of the synthesized method are time-saving and low energy consumption.

### Physical properties

**Figure 2:**
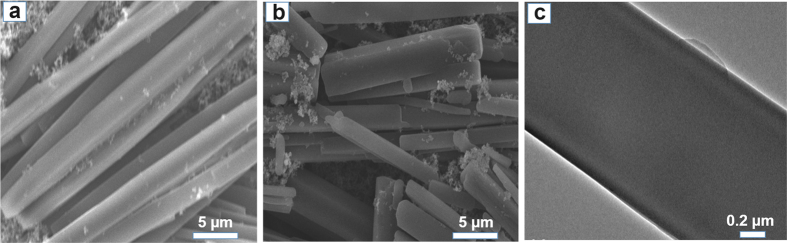
(**a**) SEM image of DP-NTCDI; (**b**) SEM image of DP-NTCDI-250; (**c**) TEM image of DP-NTCDI.

The as-prepared DP-NTCDI and DP-NTCDI-250 were comparatively characterized against each other by various techniques. From the scanning electron microscopy (SEM) image in Fig. 2a, DP-NTCDI consists of uniform rod structure, and the mean diameter of DP-NTCDI is estimated to be ~2 μm. After heat treatment of DP-NTCDI, the morphology of DP-NTCDI-250 is almost same with DP-NTCDI (Fig. 2b). The transmission electron microscopy (TEM) image in Fig. 2c manifests that DP-NTCDI is well faceted and have very smooth surface with diameter of ~2 μm.

**Figure 3:**
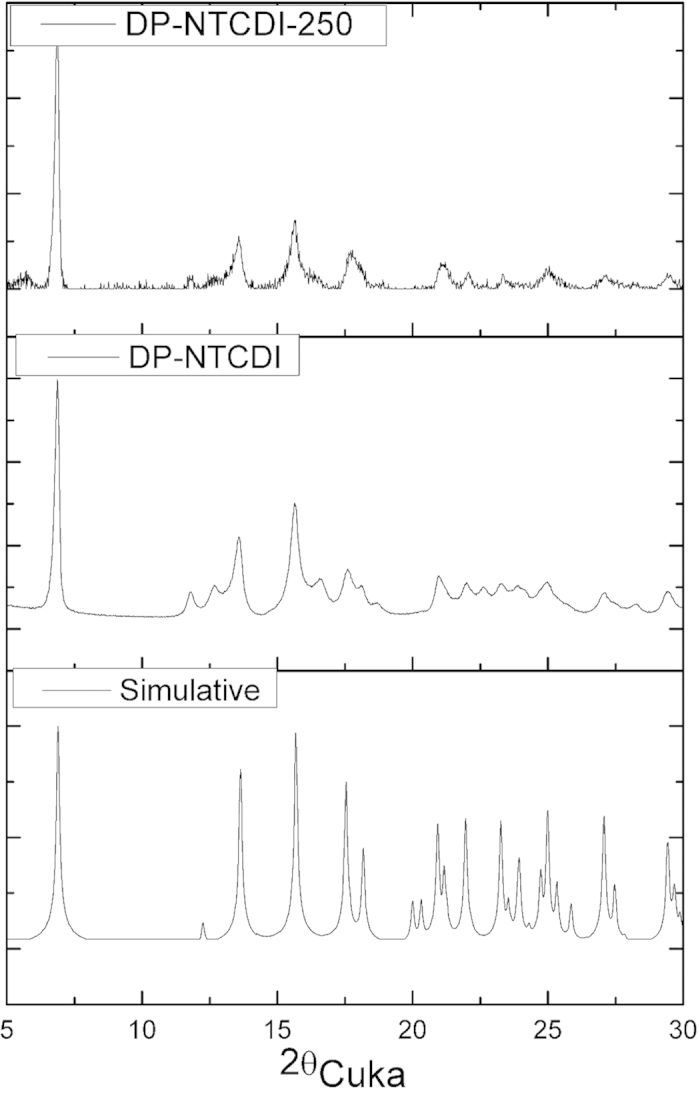
XRD patterns of DP-NTCDI-250, DP-NTCDI and simulative DP-NTCDI of ref.^35^.

X-ray diffraction (XRD) patterns in Fig. 3 are performed to investigate the crystal phase composition of DP-NTCDI and DP-NTCDI-250. It can be seen from Fig. 3 that most of the peaks from the experimental XRD patterns of DP-NTCDI and DP-NTCDI-250 can match with the simulated patterns based on single-crystal X-ray solution^35^, indicating that the ordered material is similar in the structure. All the diffraction peaks of DP-NTCDI could be indexed to monoclinic lattice of space group P2_1_/c with the lattice constants: a = 5.136 A˚; b = 7.522 A˚; c = 25.623 A˚. After heat treatment of sample, the characteristic peaks of DP-NTCDI-250 become sharply and strong. However, the characteristic peaks of DP-NTCDI and DP-NTCDI-250 are overall broad and weak in comparison with the simulated peaks, indicative of the poor crystallinity of as prepared materials.

Thermogravimetric (TG) analysis was conducted to follow the heat treatment process of DP-NTCDI and DP-NTCDI-250 in air (Fig. S1, ESI^†^). The obtained results show no weights lost were observed before 380 °C for DP-NTCDI and DP-NTCDI-250, exhibiting good thermal stability for the two samples. Upon further heating, the samples start to decompose and the framework collapses.

**Figure 4:**
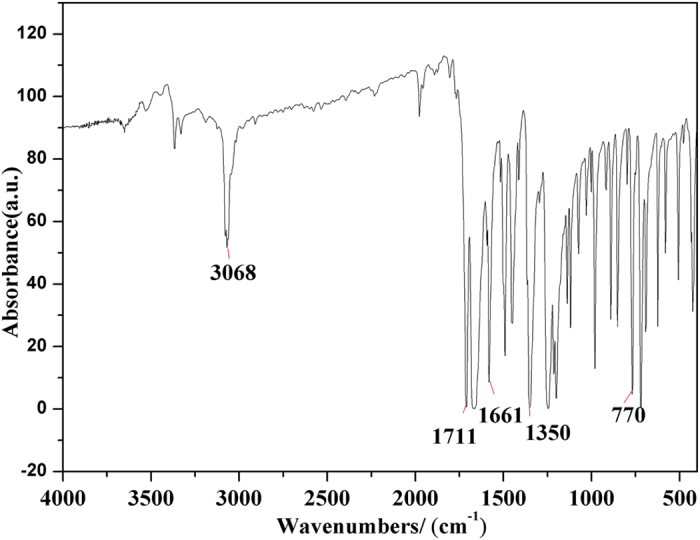
FTIR of DP-NTCDI-250.

DP-NTCDI is further supported by FTIR, ^1^H NMR and ^13^C NMR. As shown in Fig. 4, the typical absorption band at around 1711, 1661 and 770 cm^−1^ are attributed to the stretching vibration of C=O, 3068 and 982 cm^−1^ is the vibration C-H of aromatic ring, 1670 and 1350 cm^−1^ are the C-N stretching vibration of imide, 1582 cm^−1^ is the stretching vibration of naphthalene ring skeleton. As can be from the ^1^H NMR spectrum (Fig. S2a, ESI^†^), there is a multiplet peak at 7.46–7.59 ppm, which is the peak of proton on the benzene ring. A singlet at 8.73 ppm can be assigned to the proton of naphthalene ring. The two different environmental proton give an integration ratio of 5:2. For ^13^C NMR (Fig. S2b, ESI^†^), 163.42 ppm is the characteristic peak of C=O, the other seven characteristic peaks are attribute to carbon on the aromatic ring.

### Electrochemical Performance

**Figure 5:**
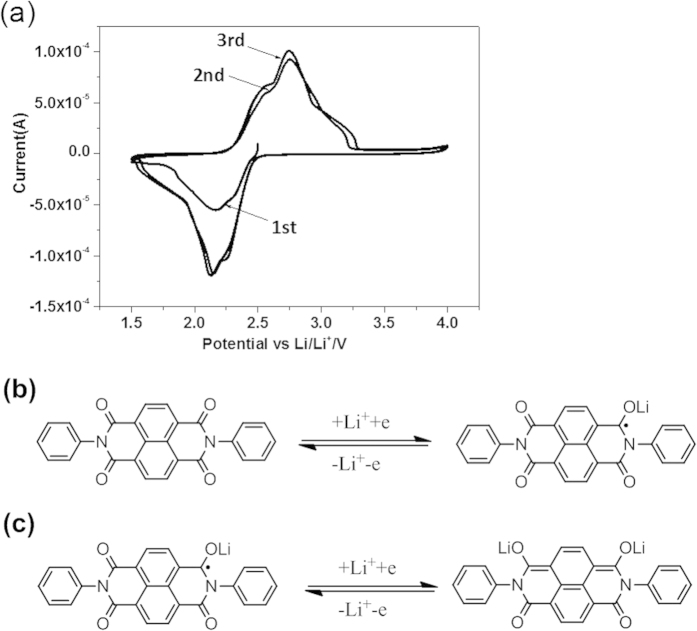
(**a**) Cyclic voltammograms of DP-NTCDI-250, scan rate: 0.1 mV s^−1^; (**b**) schematic diagram for the reversible Li-ion insertion/de-insertion in DP-NTCDI-250.

The initial three cyclic voltammograms (CVs) of DP-NTCDI electrode between 1.5 and 4.0 V at a scan rate of 0.1 mV s^−1^ is shown in Fig. 5. It can be seen from Fig. 5a that two separated steps for lithium insertion and removal in DP-NTCDI are not obvious. The first reduction peaks at about 2.3 V corresponds to one-electron transfer reaction in one of the four carbonyl groups, forming the radical-anion (the lithium enolate). The second reduction peaks at about 2.1 V can be ascribed to the second electron transfer reaction in another of the four carbonyl groups and produce a dianion species. During the positive sweeping, the two indistinct split oxidation peaks at about 2.5 V and 2.7 V are attributed to the reoxidation of lithium enolate to form the carbonyl groups^20^,^39^. The reaction processes of DP-NTCDI can be expressed as enolization process of the carbonyl group (Fig. 5b,c). DP-NTCDI comprising of four carbonyl groups makes it possible for transfering four electrons. However, the lithium enolate of only two carbonyls can be involved in DP-NTCDI when the discharge voltage is higher than 1.5 V^8^,^39^. It is worth mentioning that DP-NTCDI with conjugated structure can disperse charge by delocalization, facilitating the enolization process of the carbonyl group^8^,^39^.

**Figure 6:**
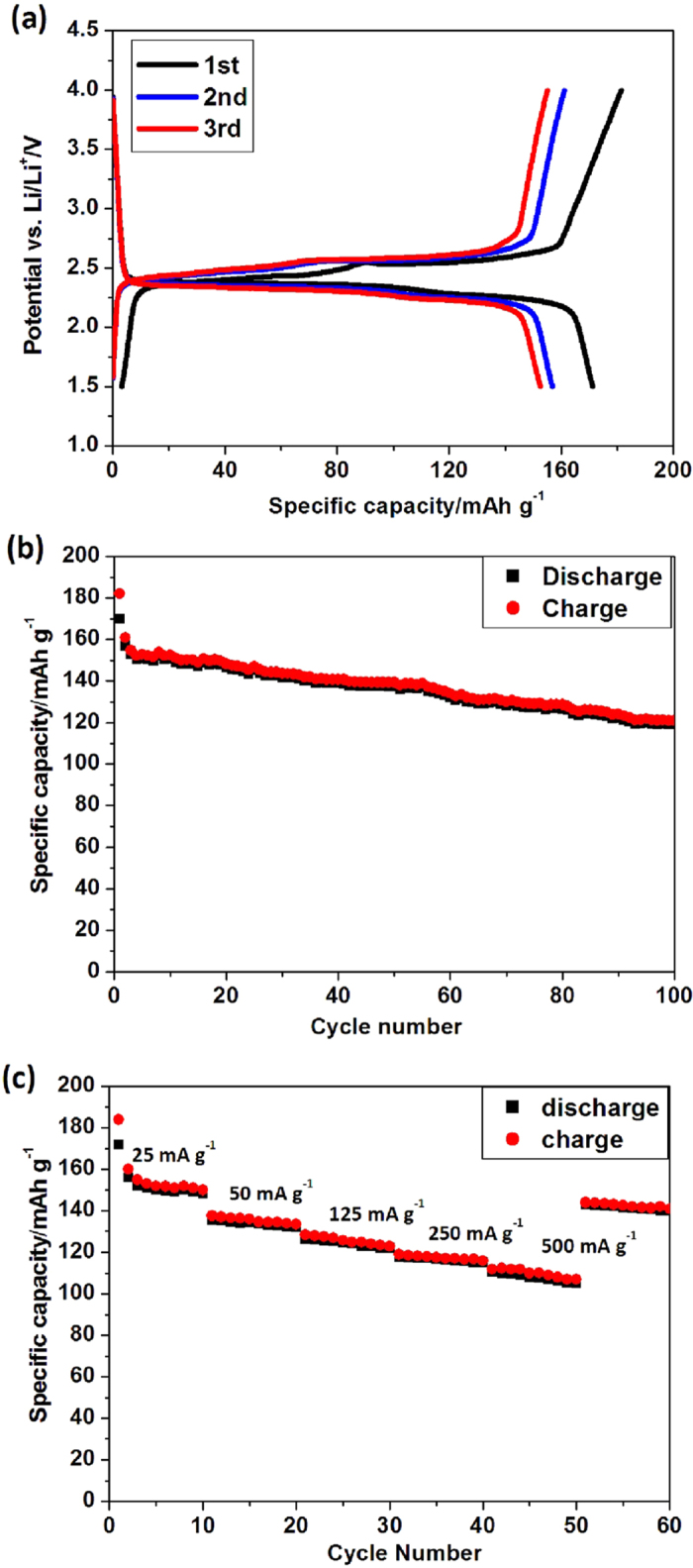
(**a**) The initial three cycles discharge-charge curves of DP-NTCDI-250 (current density: 25 mA g^−1^); (**b**) The cyclic performance of DP-NTCDI-250 (current density: 25 mA g^−1^); (**c**) The rate capabilities of DP-NTCDI-250.

The galvanostatic charge/discharge tests of DP-NTCDI-250 electrode were carried out in a potential range of 1.5–4.0 V at different current rates (Fig. 6). Figure 6(a) shows the initial three cycles for DP-NTCDI-250 electrode at a current density of 25 mA g^−1^. The battery exhibited two discharge plateaus with average discharge voltage of ~ 2.4 and ~2.3 V, respectively, corresponding to two major redox reactions observed in cyclic voltammetry. The initial discharge and charge capacity of DP-NTCDI-250 electrode is 170 and 182 mAh g^−1^, with a coulombic efficiency of 93.4%, which is higher than similar structures in literatures^37^,^38^. After three cycles, the reversible capacity of DP-NTCDI-250 electrode is ~156 mAh g^−1^, with the coulombic efficiencies of DP-NTCDI-250 over 98%. However, the initial charge capacity of the material corresponds to 87% of theoretical capacity (256 mAh g^−1^), which still cannot reach its full theoretical capacities, it may be ascribed to the low crystallization and the poor electronic conductivity of organic compounds. Figure 6(b) shows the cycle performance of DP-NTCDI-250 at a current rate of 25 mA g^−1^. After 100 cycles, the reversible capacities of DP-NTCDI-250 electrode maintains ~120 mAh g^−1^ with the capacity fading of 13%. This result shows that DP-NTCDI-250 present better cycle life compared with those similar structures that have been reported in literature^37^,^38^,^39^, which might be attributed to bigger particle size and rod-like structure^30^. The rate capability of the sample is evaluated at different rates from 25 to 500 mA g^−1^ (Fig. 6(c)). It can be found from Fig. 6(c) that DP-NTCDI-250 exhibits good rate capability with reversible capacities of 153, 134, 127, 118, 109 mAh g^−1^ at current densities of 25, 50, 125, 250, 500 mA g^−1^ (each for 10 cycles), respectively. After 50 cycles, the reversible capacity still maintains 106 mAh g^−1^. When the current density back to 25 mA g^−1^, the reversible capacity is 142 mAh g^−1^, demonstrating good rate capacity and reversibility.

To explain the well electrochemical performance of DP-NTCDI-250, electrochemical impedance spectroscopy (EIS) was performed. Figure S3 shows the Nyquist plots of DP-NTCDI-250 electrode before cycling and after 100 cycling at 25 mA g^−1^. It can be found that the diameter of semicircle after cycling is basically same with that of before cycling, demonstrating that the charge transfer resistance of DP-NTCDI-250 electrode after cycling basically did not increase and the structure was not damaged, indicating the good stability of the active materials. The good cycling performance of DP-NTCDI-250 at 25 mA g^−1^ were further confirmed by SEM and the dissolution test of the sample (Figs S4 and S5). Figure S4 presents the SEM image for DP-NTCDI-250 electrode after cycling. As can be seen from Fig. S4, after 100 cycles, although the size of rod-like structure becomes small, the morphology of rod-like structure of DP-NTCDI-250 electrode can be maintained and can’t be damaged, contributing the good cycling stability of DP-NTCDI-250 electrode. Figure S5 shows the photos of DP-NTCDI-250 dissolution in electrolyte. It can be seen that DP-NTCDI-250 sample almost can’t be dissolved in electrolyte after 15 days, implying DP-NTCDI-250 can achieve good electrochemical performance.

## Conclusion

DP-NTCDI-250 sample consisting of rod-like structure was synthesized through a simple one-pot *N*-acylationreaction of 1,4,5,8-naphthalenetetracarboxylic dianhydride (NTCDA) with phenylamine (PA), leading to great time saving compared with the other organic synthesized method. The electrochemical tests including constant current charge-discharge, cyclic voltammetry (CV), and electrochemical impedance spectroscopy (EIS) show high specific capacity, good cycling stability, high-rate capability for DP-NTCDI-250 compound. The synthesis process is simple, time saving and low energy consumption. The well electrochemical performance is also promising for the application in lithium batteries.

## Methods

1,4,5,8-Naphthalenetetracarboxylic dianhydride (NTCDA), phenylamine (PA), triethylamine (Et_3_N), *N*,*N*′-dimethylformanide (DMF), ethanol (EtOH) were obtained from commercial sources and used without purification.

### Preparation of *N,N’*-diphenyl-1,4,5,8-naphthaleetetracarboxylic diimide (DP-NTCDI)

*N,N′*-diphenyl-1,4,5,8-naphthalenetetracarboxylic diimide (DP-NTCDI) was prepared by 1,4,5,8-naphthalenetetracarboxylic dianhydride (NTCDA) with phenylamine (PA) in DMF solution. Typically, 2.0 g NTCDA (7.6 mmol) was dissolved in 40.0 mL DMF at 130 °C under stirring. Then 2.1 mL PA (23.0 mmol) and 4.3 mL of Et_3_N (30.0 mmol) were added dropwise into the solution, respectively, and continued to reflux under stirring for 1 hour to form a brown precipitate, and the mother liquid was removed by centrifugation and filtration. The as-obtained brown precipitate was redissolved in EtOH, washed thoroughly with EtOH. Then the as-obtained product with recrystallization in DMF afforded orange acicular DP-NTCDI. The as-obtained pure DP-NTCDI was washed with EtOH and collected by filtration again. Finally, the pure DP-NTCDI was dried in vacuum at 80 °C for 12 h (2.9 g, 91.2%): ^1^H NMR (DMSO) δ 8.73 (s, 4H), 7.46–7.59 ppm (m, 10H); ^13^C NMR (DMSO) δ 127.14, 127.45, 128.94, 129.43, 129.47, 130.88, 136.06, 163.42 ppm. IR (KBr pellets): v = 3068, 981.77(benzene C-H), 1710.86 (C=O, vas), 1670.35 (imide), 1660.71 (C=O, vs), 1581.63 (naphthalene C=C, v), 1350.17 (C-N, v), 769.60 cm^−1^ (C=O, δ). The elemental analysis by a Thermo Flash EA-1112 (CHNS-O) element analyzer indicated that the synthesized DP-NTCDI consists of 74.60% C, 3.32% H, 6.62% N and 15.36% O, corresponding to its calculated values: 74.64% C, 3.37% H, 6.70% N and 15.30% O.

The as-prepared DP-NTCDI was further annealed at 250 °C for 3 h under air atmosphere to obtain DP-NTCDI-250 sample.

### Characterization

The morphology and structure of as-prepared DP-NTCDI and DP-NTCDI-250 were characterized by a field-emission scanning electron microscopy (FESEM, ZEISS Ultra 55, 5 kV, Pt-spraying treatment), transmission electron microscopy (TEM, JEM-2100HR, 200 kV), powder X-ray diffraction (XRD, BRUKER D8 ADVANCE, Cu K radiation (1.5406)). Elemental analyses were performed on a Thermo Flash EA-1112 (CHNS-O) element analyzer. The FT-IR spectra were recorded from KBr pellets in the 4000 ~ 400 cm^−1^ range on a Shimadzu Fourier Transform Infrared Spectrometer (IR Prestige-21). Thermogravimetric analyses (TGA) were carried out on a Perkin-Elmer TGA 7 thermo-gravimetric analyzer. ^1^H and ^13^C NMR spectra were obtained in DMSO-d_6_ on a Varian VNS400 MHz spectrometer and tetramethylsilane (TMS) was used as internal standard.

### Electrodes and Cells Fabrication

Coin-type cells (size:2016) consisting of a working electrode and a lithium foil counter electrode separated by a Celgard 2300 microporous membrane were assembled in an argon-filled glove-box. The working electrodes were prepared by mixing a powder of DP-NTCDI-250, acetylene black as a conductive additive, and polyvinylidene fluoride (PVDF) as a binder in a weight ratio of 5:4:1 using a mortar. The sheet was then attached to an aluminum foil current collector, and the resultant working-electrode was dried. The amount of active material deposited was approximately 3 mg cm^−2^. The electrolyte was 1M LiPF_6_-EC + EMC + DMC (V_EC_:V_EMC_:V_DMC_ = 1:1:1). The charge/discharge experiments were performed by using a LAND cell test system (Land CT 2001A) in the potential range of 1.5~4.0 V versus Li/Li^+^ at different constant current densities (25, 50, 125, 250, 500 mA g^−1^). Cyclic voltammogram was obtained on PGSTAT-30 (Autolab) in the potential range of 1.5~4.0 V at a scanning rate of 0.1 mV s^−1^. The electrochemical impedance spectroscopy (EIS) analysis were carried in the frequency range from 100 kHz to 10 mHz with an AC signal amplitude of 10 mV.

## Additional Information

**How to cite this article**: Lv, M. *et al.* Heteroaromatic organic compound with conjugated multi-carbonyl as cathode material for rechargeable lithium batteries. *Sci. Rep.* **6**, 23515; doi: 10.1038/srep23515 (2016).

## Supplementary Material

Supplementary Information
