# Supplementary material for: Heteroaromatic organic compound with conjugated multi-carbonyl as cathode material for rechargeable lithium batteries
Source: Sci Rep. 2016 Apr 11;6:23515. doi: 10.1038/srep23515 (PMC4827395; doi:10.1038/srep23515)
Supplement: Supplementary Information [file srep23515-s1.doc]

**Supplementary Information**

Subject areas: Energy science and technology

Correspondence and requests for materials should be addressed to R. Zeng (email: [zengronghua@m.scnu.edu.cn](mailto:zengronghua@m.scnu.edu.cn)), H. Zeng ( email: zenghp@scnu.edu.cn ) and S. -L. Chou (email: [shulei@uow.edu.au](mailto:shulei@uow.edu.au))

**Heteroaromatic organic compound with conjugated multi- carbonyl as cathode material for rechargeable lithium batteries**

Meixiang Lv^1^, Fen Zhang^1^, Yiwen Wu^1^, Mujuan Chen^1^, Chunfeng Yao^2^, Junmin Nan^1^, Dong Shu^1^, Ronghua Zeng^1*^, Heping Zeng^1*^, Shu-Lei Chou^3*^

^1^School of Chemistry and Environment, Guangzhou Key Laboratory of Analytical Chemistry for Biomedicine, South China Normal University, Guangzhou 510006, China.

^2^Research Resources Center, South China Normal University, Guangzhou 510006, China.

^3^Institute for Superconducting and Electronic Materials，Australian Institute for Innovative Materials, University of Wollongong, Innovation Campus, Squires Way, North Wollongong NSW 2522, Australia.


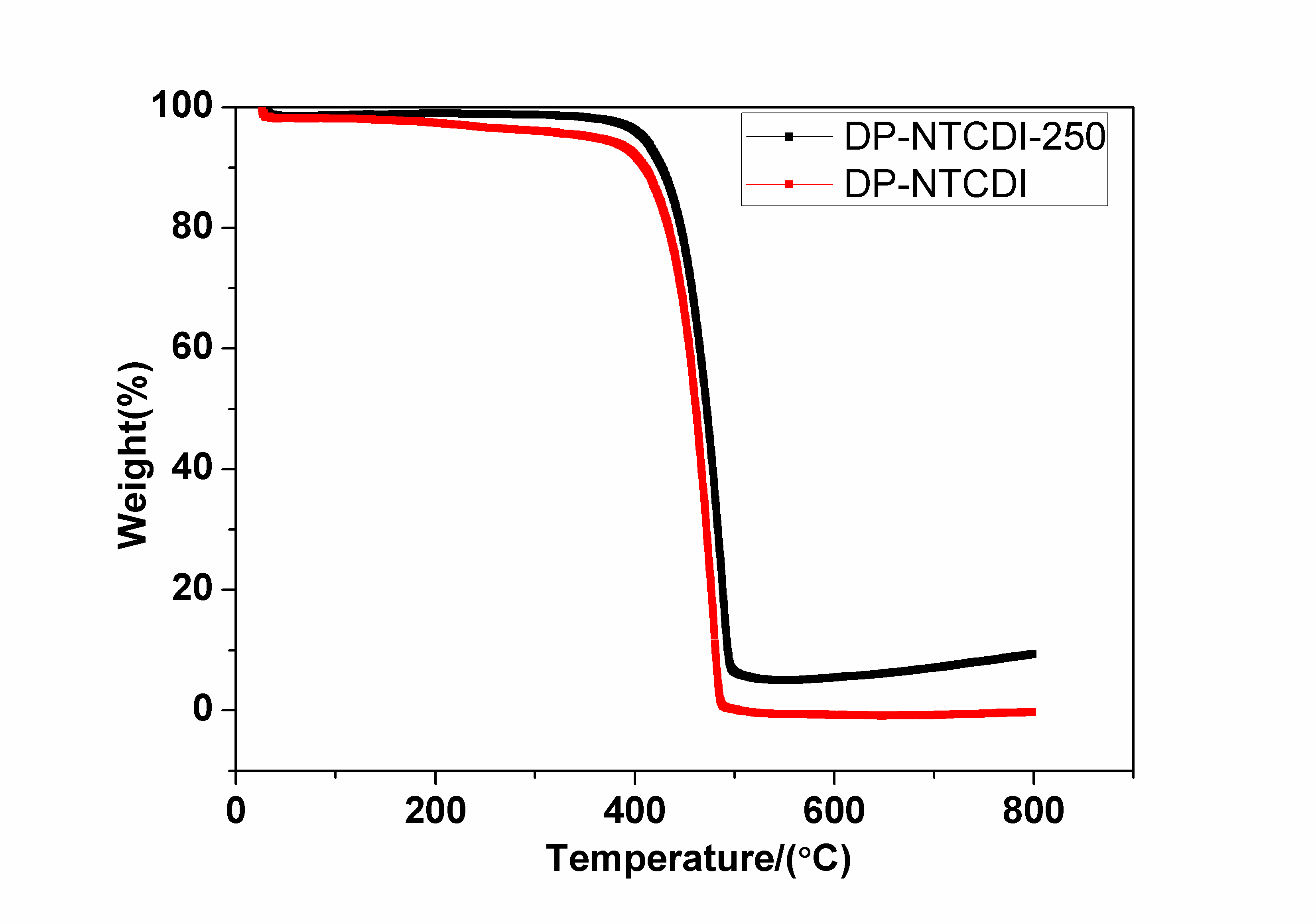


**Figure S1.** TGA curves of DP-NTCDI and DP-NTCDI-250.

.


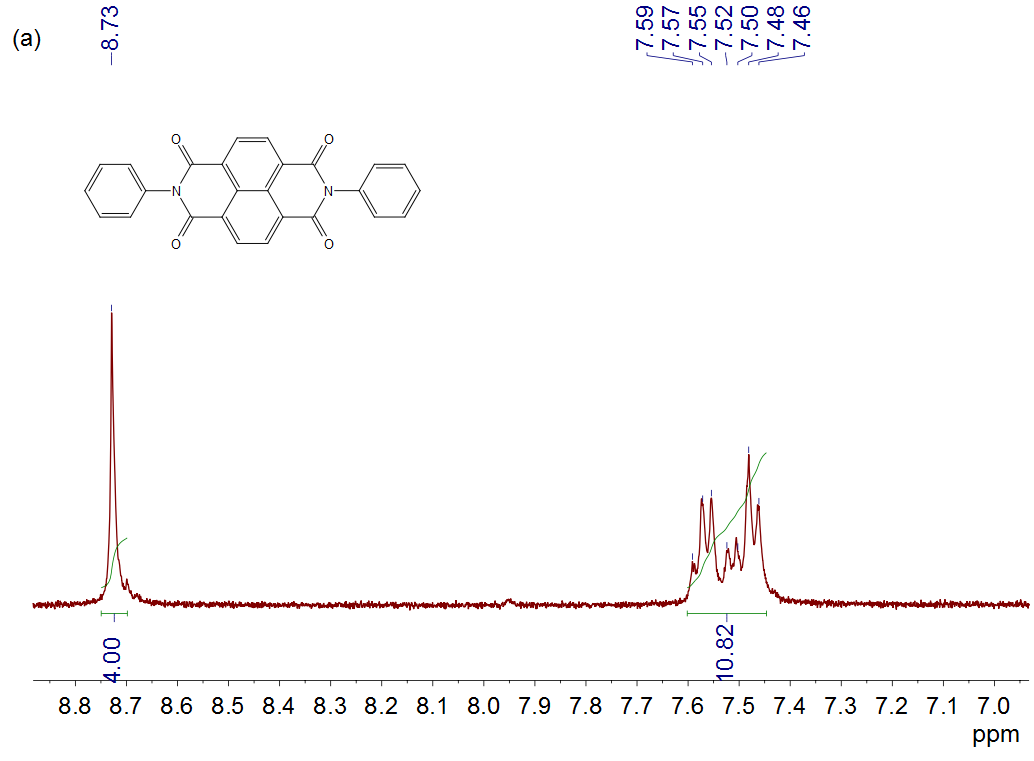


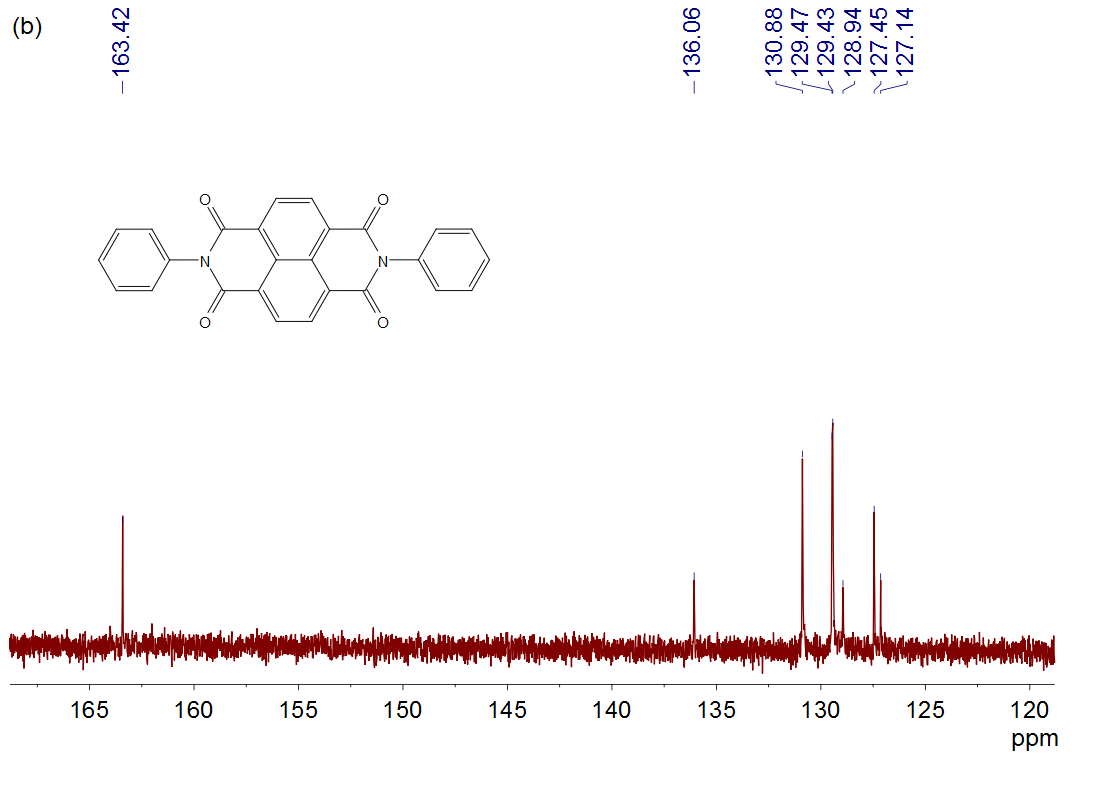


**Figure S2.** (a) ^1^H NMR spectra of DP-NTCDI, (b) ^13^C NMR spectra of DP-NTCDI.


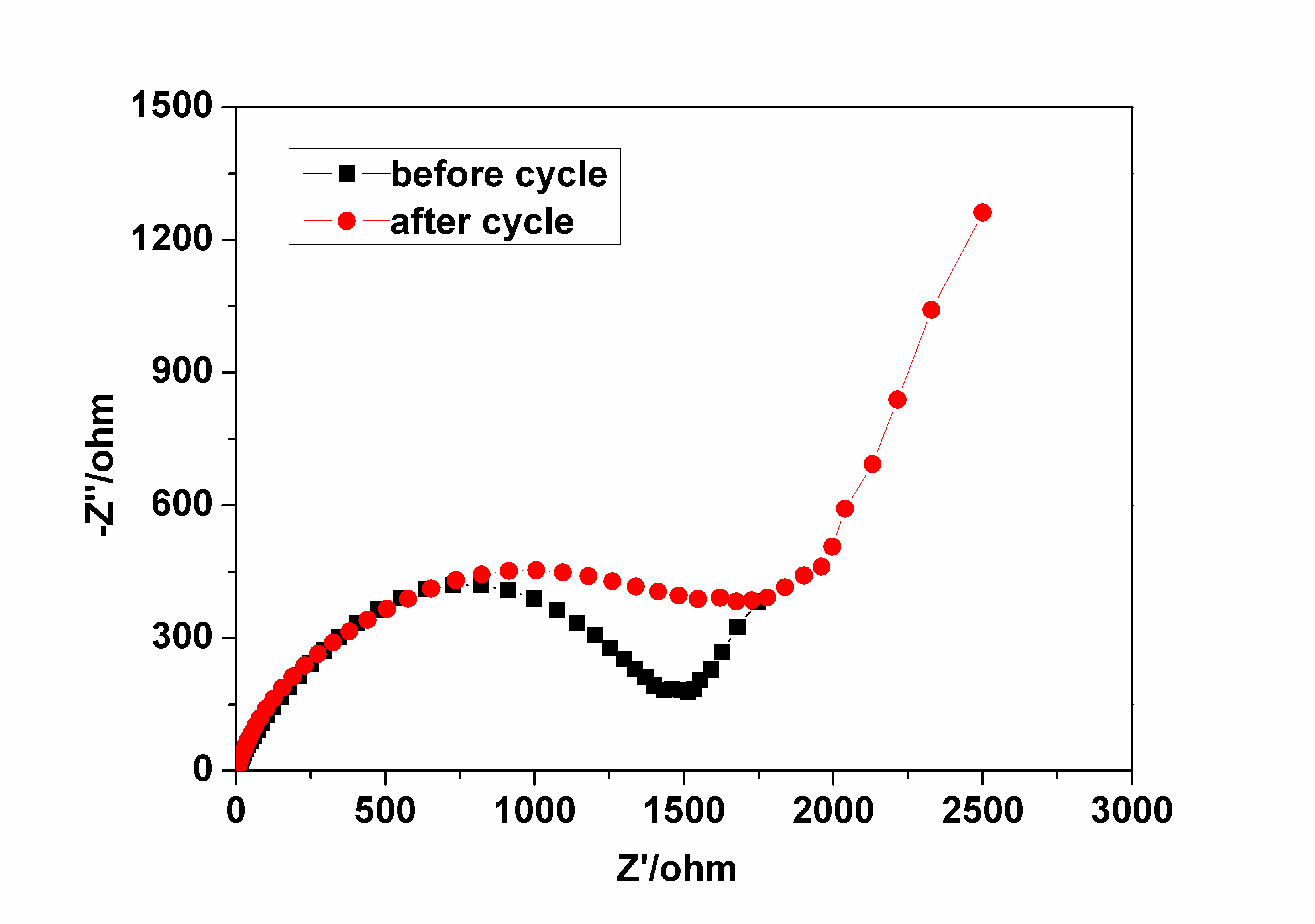


**Figure S3.** Nyquist plots of DP-NTCDI-250 before cycle and after cycle.


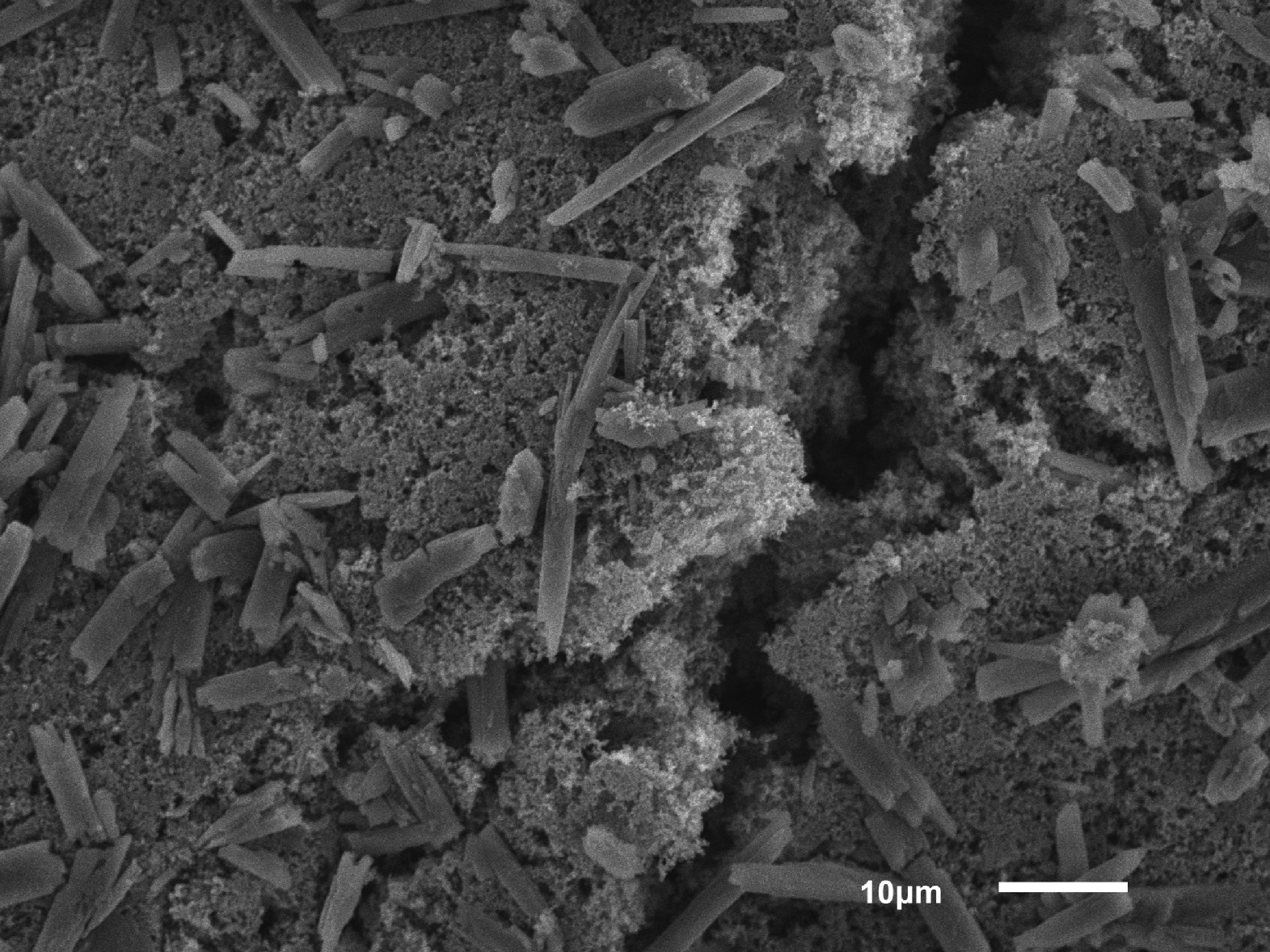


**Figure S4.** The image of DP-NTCDI-250 after 100 cycles.


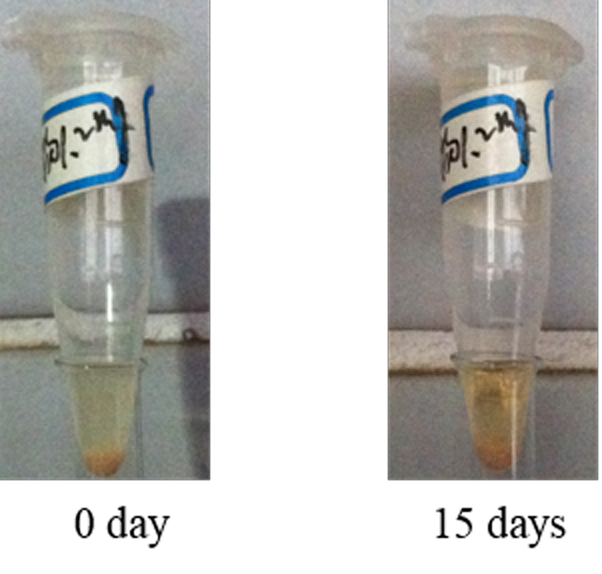


**Figure S5.** The photos of dissolution experiment of DP-NTCDI-250 in electrolyte.
